# Qualitative development of the daily identity‐related stress and resilience model using daily diaries from LGBTQ+ youth in the southeastern United States

**DOI:** 10.1111/jora.70289

**Published:** 2026-10-20

**Authors:** Kaitlyn Phillips, Alexandros Nikolaidis‐Konstas, Eric Layland, Kirsty Clark

**Affiliations:** ^1^ Department of Medicine, Health, and Society Vanderbilt University Nashville Tennessee USA; ^2^ Department of Psychology and Human Development Vanderbilt University Nashville Tennessee USA; ^3^ Department of Psychology Harvard University Cambridge Massachusetts USA; ^4^ Department of Human Development and Family Sciences University of Delaware Newark Delaware USA

**Keywords:** daily diary, ecological momentary assessment, intensive longitudinal methods, LGBTQ+ youth, minority stress, resilience, socioecological levels

## Abstract

The prevailing reliance on cross‐sectional methods in LGBTQ+ mental health research has limited researchers' ability to capture the dynamic, multilevel processes of minority stress and resilience factors as they unfold over time. Although socioecological frameworks have been applied longitudinally, they have rarely attended to the interplay of historical, developmental, and short‐term timescales that shape LGBTQ+ youth's day‐to‐day experiences. Using qualitative daily diary data from a smartphone‐based ecological momentary assessment (EMA) study, we pursued two aims: (1) to characterize LGBTQ+ youth's daily positive and negative identity‐related experiences using qualitative content analysis; and (2) to develop a conceptual model illustrating how minority stress and resilience factors co‐occur and unfold within daily life. Over 28 consecutive days, 50 LGBTQ+ youth aged 13–24 (*M*_age_ = 18.38, SD = 3.02; 58% transgender or gender‐diverse) living in the U.S. Southeast and at elevated risk for suicide completed nightly diary entries describing the most positive and most negative event of their day. Qualitative content analysis of 349 identity‐related diaries (108 negative, 241 positive) revealed that minority stress and resilience factors co‐occurred across structural, interpersonal, and intrapersonal socioecological levels within single days, and that identity‐related experiences showed meaningful continuity across days at the within‐person level. Drawing on these findings and existing socioecological frameworks of minority stress, we developed the *Daily Identity‐Related Stress and Resilience Model*, a conceptual framework that positions stress and resilience factors as simultaneous, multilevel processes embedded within individuals' daily lives and shaped by time‐stable social and identity factors across both short‐ and long‐term timescales. Implications for future research and intervention development with LGBTQ+ youth are discussed.

## INTRODUCTION

Lesbian, gay, bisexual, transgender, and other queer (LGBTQ+) youth experience poorer mental health outcomes—such as depression and suicidal thoughts and behaviors—than their cisgender and heterosexual peers; disparities which have been well‐documented in systematic reviews (O'Shea et al., 2025; Wilson & Cariola, 2020) and meta‐analyses (di Giacomo et al., 2018; Marshal et al., 2011). Much of the existing research into factors potentiating poorer mental health among LGBTQ+ youth has examined minority stressors (e.g., discrimination, victimization) as the primary explanatory factors, overlooking other critical facets of youth well‐being, such as strengths‐based perspectives (Fish, 2020; Vaughan & Rodriguez, 2014). Additionally, most studies on LGBTQ+ mental health rely on cross‐sectional data (Williams et al., 2021), limiting the ability to capture time‐varying stress and resilience processes, as well as their links with mental health, as they unfold over time. Recent advances in intensive longitudinal data collection offer opportunities to document daily experiences of stress, resilience, and mental health outcomes (Clark et al., 2024; Flynn et al., 2024), but these methods have been underutilized in research with LGBTQ+ youth. Real‐time qualitative data, such as daily diaries, can further illuminate associations between stress and resilience processes, providing deeper insights into their interconnection in the daily lives of LGBTQ+ youth.

In this study, we integrate theoretical literature—including minority stress and strengths, socioecological systems theory, and the role of attending to time in stigma research—with daily diary data from LGBTQ+ youth in a high‐stigma region of the United States to examine daily identity‐related stressors and resilience experiences as they unfold in everyday life. Drawing on these data, we propose a new conceptual model of *Daily Identity‐Related Stress and Resilience*, which we hope will serve as a foundation for future research and intervention development. Below, we review existing literature on minority stress/strengths and socioecological systems theory as they relate to LGBTQ+ youth. We also highlight the importance of intensive longitudinal data in advancing insights on mental health, stress, and resilience in this population.

### The minority stress model and strengths‐based considerations

The minority stress model, initially proposed by Brooks (1981) and advanced by Meyer (2003), has been pivotal in the conceptualization of factors contributing to LGBTQ+ health inequities. The model proposes that minority stressors constitute additional stress above‐and‐beyond the general stress that all people experience, which thereby increases the risk of negative health outcomes for LGBTQ+ populations relative to the general population (Frost & Meyer, 2023). Stressors can be distal (i.e., external) or proximal (i.e., internal). Distal stressors are either interpersonal (e.g., victimization) or structural in nature (e.g., discriminatory policies), while proximal stressors operate intrapersonally (e.g., internalized stigma; Frost & Meyer, 2023). Exposure to minority stress is also theorized to increase maladaptive stress responses—including adverse social, biological, or psychological processes—that can exacerbate poor health outcomes (Hatzenbuehler, 2009). Although the original minority stress model was developed with sexual minority people in mind, it has since been extended to include the experiences of transgender and gender‐diverse people (Hendricks & Testa, 2012; Lefevor et al., 2019). A well‐established body of research has shown that minority stressors are associated with negative mental health outcomes, such as substance use (Lehavot & Simoni, 2011) and suicidality (de Lange et al., 2022). The framework has also been adapted to better capture youth perspectives. For example, Goldbach and Gibbs (2017) developed an adolescent‐focused adaptation of the minority stress model, which highlighted possible developmentally appropriate amendments, including the importance of considering young people's social contexts (e.g., family and school), the presence of coping resources (e.g., supportive religious community), and considerations about identity development (e.g., stress about identity formation).

A criticism that has been voiced in regard to the minority stress model is that it has primarily been leveraged in a deficit‐oriented manner that neglects resilience in the lives of LGBTQ+ people, contributing to the proliferation of negative views about LGBTQ+ health (Colpitts & Gahagan, 2016; Peel et al., 2023). Developmental conceptualizations of resilience define it as a dynamic process of positive adaptation in the context of adversity, shaped by protective factors operating across individual, interpersonal, and contextual levels rather than as a fixed individual trait (Luthar et al., 2000; Masten, 2014; Zimmerman et al., 2013). In response to such criticisms, researchers have argued that pathology and resilience are not mutually exclusive (Meyer, 2014), suggesting that protective factors may facilitate resilient adaptation in the context of minority stress while cautioning against deflecting the focus from institutional barriers to personal strengths of LGBTQ+ people. The importance of resilience at different levels within the minority stress framework has also been emphasized, including resilience at the structural level, which refers to the ways in which communities at broader levels (e.g., national, local) foster their members' well‐being (Meyer, 2015).

In response to concerns about the adoption of a deficit‐oriented lens, researchers have developed the minority strengths model to complement and extend the minority stress framework (Perrin et al., 2020). This model proposes that strength‐based protective factors such as identity pride, self‐esteem, and community consciousness promote resilience and positive health outcomes. The minority strengths model has recently been utilized to explore nuances of LGBTQ+ experiences that encompass both stress and resilience frameworks, such as cannabis use as a means of building community among queer and trans youth in Quebec (London‐Nadeau et al., 2024). In sum, while the minority stress model remains the most prevalently applied theoretical framing of LGBTQ+ youth health, researchers have begun calling for newer frameworks—such as minority strengths—that explicitly integrate protective factors and unique, favorable LGBTQ+ qualities to provide a fuller conceptualization that recognizes the interplay of LGBTQ+ youth stress and strengths (Fish, 2020; Vaughan & Rodriguez, 2014).

### Socioecological systems theory and its relation to LGBTQ+ stress and resilience

Central to the exploration of stress and resilience is the socioecological systems theory, which was originally conceived as a developmental model aimed at illustrating the ways in which different systems impact children's development (Bronfenbrenner, 1979). The theory, which has since been reconceptualized as the bioecological systems theory (Bronfenbrenner & Ceci, 1994; Tong & An, 2024), suggests that youth are at the center of multiple nested, reciprocally interacting systems related to intrapersonal, interpersonal, and broader structural processes. The outermost of these systems—the chronosystem—posits that changes in the environment or the youth over time are integral to development (Bronfenbrenner, 1994). The socioecological systems theory has informed the way in which researchers view stressors, such as bullying and victimization (Hong & Espelage, 2012), as experiences within nested systems. Simultaneously, it has inspired a deeper conceptualization of resilience within the developmental literature, suggesting that youth's ability to effectively navigate adversity—including stressful experiences—is not solely bound to individual resilience, but also to the quality of their interactions with different nested systems and the resources these systems provide (Ungar et al., 2013).

Driven by the socioecological systems theory adaptations (McLeroy et al., 1988) and informed by the multilevel nature of LGBTQ+ stigma (Hatzenbuehler & Pachankis, 2016; van der Star, 2024), experts often conceptualize LGBTQ+ stress and resilience within the following socioecological systems: *intrapersonal* (also referred to as *individual* in the literature; e.g., internalized stigma), *interpersonal* (e.g., victimization and discrimination), and *structural* (e.g., discriminatory laws and policies). The application of this framework is flexible and not always consistent in the literature; some researchers disaggregate levels into additional systems, such as by distinguishing community (e.g., non‐affirming religious community; Emetu & Rivera, 2018) from interpersonal and structural levels. Among transgender populations, the socioecological systems theory has been instrumental in allowing researchers to posit the ways in which stigma at the intrapersonal (e.g., internalization of transgender stigma), interpersonal (e.g., discriminatory healthcare interactions), and structural (e.g., stigmatizing policies) levels impact health and inform appropriate, targeted interventions (White Hughto et al., 2015). Similarly, the framework has been extended to conceptualize minority stress as composed of multilevel stressful experiences for parents of LGBTQ+ youth in the U.S. Southeast (e.g., fear of stigmatization; Argiros et al., 2024). Although the use of the socioecological systems theory has primarily been tied to stress and harm for LGBTQ+ youth, a systematic review highlighted resilience factors across the systems for transgender and gender‐diverse youth health, emphasizing, for instance, the importance of school visibility and peer support (Johns et al., 2018). In sum, this theoretical framework has been pivotal in allowing experts to conceptualize the multilevel nature of LGBTQ+ stress and resilience, while drawing connections to the minority stress literature.

Formal attempts to synthesize the socioecological systems and the minority stress model demonstrate the potential to integrate these distinct theories into a more comprehensive framework. For example, van der Star (2024) proposed that the effects of LGBTQ+ stigma on health can be represented by three main pillars: (i) the chronosystem, (ii) the nested socioecological systems (i.e., intrapersonal, interpersonal, structural), and (iii) the mechanisms (i.e., social, biological, or psychological processes; based on Hatzenbuehler, 2009) that mediate or moderate the effects of stigma on health outcomes. While the suggested model posits that bidirectional relationships may exist between the socioecological systems, it argues that the direction of stigma primarily flows from the structural to the interpersonal and, finally, the intrapersonal level. Similarly, Lee et al. (2024) used the original ecological systems theory (Bronfenbrenner, 1994) to model minority stress and resilience as separate downward cascades from the outermost system (i.e., the chronosystem) to the intrapersonal level. Much like the framework proposed by van der Star (2024), the authors acknowledge that smaller systems (e.g., the intrapersonal) may potentially affect larger systems (e.g., structural). In addition, they suggest that resilience and stress may be related, despite being represented as distinct processes. Taken together, these recent theoretical advances highlight the importance of conceptually modeling the multilevel nature of LGBTQ+ minority stress and strengths within ecological systems to better explain LGBTQ+ health processes.

### The importance of time and the promise of intensive longitudinal data

Recent research posits that considering time is essential—and often overlooked—when investigating the effects of LGBTQ+ stress and resilience on health. As applied to LGBTQ+ youth, authors argue that multiple interacting timescales must be taken into account when studying the role of stigma on health (Earnshaw et al., 2022), including the historical context (e.g., structural heterosexism), developmental stage (e.g., bullying from peers in youth), and potential lifespan changes in the status of a stigmatized identity (e.g., sexual orientation identity fluidity in transgender and non‐binary youth; Katz‐Wise et al., 2024). The importance of time has also been proposed in relation to the minority stress model, as it has been argued that ontogenetic (i.e., developments occurring over time), generational (i.e., group‐specific differences over time), and historical time scales (i.e., historical events) are particularly relevant when considering the health of LGBTQ+ populations (Rivas‐Koehl et al., 2023).

Traditionally, the chronosystem (i.e., broad life course perspective of time; outermost ecological system) has been the main lens through which experts have explored stress and resilience over time; however, conceptualizing time in this research requires recognizing the multiple granular ways in which it unfolds, such as across historical time, life course time, and time between events. As an example of historical time, qualitative research suggests that previous experiences of marginalization and isolation may have rendered LGBTQ+ adults more resilient during the COVID‐19 pandemic (Gonzalez et al., 2021). Regarding life course time, a study analyzing data from a probability sample of sexual minority people in the United States, including youth, found that the majority of suicidal thoughts and behaviors emerged close to the time of one's realization of their sexual minority status, suggesting that this developmental milestone may coincide with minority stress experiences that negatively affect mental health (Meyer et al., 2021). Extending to recent developmental science, Russell and Fish (2019) describe a “developmental collision” in which rapid gains in LGBTQ+ visibility and acceptance over recent decades intersect with an earlier age of coming out, such that LGBTQ+ youth today encounter minority stressors that compound the already‐challenging transitions of early and middle adolescence—including puberty, shifting peer dynamics, and increasing academic and social demands. In line with minority stress research emphasizing the role of social context (e.g., family, school, peers, online and social media, religion, and LGBTQ+ community connectedness; Goldbach & Gibbs, 2017), these findings collectively underscore that broader contextual factors continue to shape, and at times exacerbate, mental health disparities among LGBTQ+ youth. In terms of event time, a recent qualitative study on transgender adults from Australia highlighted that there is dissonance in temporality between participants' expectations of when trans‐affirming care should be provided and when it is accessible by health institutions—a structural‐level stressor—resulting in psychological distress (Grant et al., 2025). Taken together, these findings suggest that time is an essential and multifaceted component that is embedded in LGBTQ+ stress, resilience, and health, pointing to the importance of considering nuanced time scales (e.g., developmental trajectories, historical events, contemporary structural context) in LGBTQ+ health research.

In addition, studies highlighting the relationship between time and stress or resilience sometimes propose that smaller time scales also be considered when investigating within‐person time (i.e., changes occurring within an individual over time). For example, it has been suggested—but not yet formally argued—that a microscale captured through intensive longitudinal data (i.e., the repeated collection of data from a single participant over a pre‐determined period of time) may provide granular insight into the short‐term effects of stigma or resilience as they unfold (Earnshaw et al., 2022; Lee et al., 2024). Recent empirical research has explored multiple mental health outcomes in LGBTQ+ youth using such designs. For example, an ecological momentary assessment (EMA; a type of intensive longitudinal design) study on sexual minority adolescents found that minority stress exposure significantly predicted within‐person nicotine craving and negative effect (Mereish et al., 2022). Similarly, an EMA study on LGBTQ+ young adults from the U.S. Southeast found that exposure to negative LGBTQ+ news (a structural stressor) was significantly associated with within‐person increases in active and passive suicidal ideation and self‐harm ideation in the following hours (Clark et al., 2024). In terms of resilience, EMA research has found significant within‐person associations between LGBTQ+ resilience factors (e.g., identity affirmation) and positive mental health outcomes, such as increases in positive affect (Parnes et al., 2024). In sum, this nascent body of research points to the utility of intensive longitudinal designs in identifying links between stress or resilience and mental health within LGBTQ+ youth as they unfold in everyday life. It also suggests that time should not be treated monolithically as the outermost ecological system, but rather as a dimension that extends from the structural to the intrapersonal level.

Intensive longitudinal data can be advantageous in exploring LGBTQ+ stress and resilience processes that occur subtly and on smaller scales. For example, a systematic review on LGBTQ+ microaggressions—a type of stressor that is related to brief, subtle remarks that insult, stereotype, or invalidate LGBTQ+ people (Cyrus, 2017; Ramirez & Paz Galupo, 2019; Sue et al., 2007) suggests that externalizing symptoms in response to minority stress (in the form of microaggressions) may be occurring quickly after exposure and may not be adequately captured through spread‐out assessments (DeSon & Andover, 2024). Simultaneously, intensive longitudinal data allows researchers to capture momentary changes in minority stress or resilience without the need to make trait‐based assertions (e.g., resilient vs. not resilient people; Lee et al., 2024), while also alleviating concerns about recall bias and, subsequently, maximizing validity (Shiffman et al., 2008). The benefit of intensive longitudinal data collection in providing more fine‐tuned data and within‐person insights has also been explored in previous LGBTQ+ qualitative studies (e.g., Flanders et al., 2017; Flynn et al., 2024). A small—but growing—body of qualitative daily diary research has begun to document stress and resilience among LGBTQ+ youth. For example, a qualitative, daily diary study of oppression‐based stress experiences highlights how sexual and gender minority adolescents experience daily oppression‐based stressors across intrapersonal, interpersonal, and structural domains (Sullivan et al., 2024). Complementing this work, studies of resilience and positive identity processes document sources of queer and trans joy in daily life, including social support, identity exploration, and community connection (Flynn et al., 2024). Together, these studies underscore both the salience of daily stressors and the presence of affirming, resilience‐promoting experiences in LGBTQ+ youths' everyday lives. Yet, it remains critical to understand the ways in which LGBTQ+ people respond with resilience to top‐down stressors, while acknowledging the complex dynamics between stress and across nuanced time scales.

### The current study

The overarching goal of the study was to develop an applied model that is aligned with the minority stress/strengths literature and the socioecological systems, while also highlighting the unfolding of daily positive and negative LGBTQ+ experiences over brief time periods (i.e., days) among LGBTQ+ youth. Drawing on daily diary data from an EMA study of LGBTQ+ youth at elevated risk of suicide in the U.S. Southeast, we sought to: (a) apply qualitative analytic methods to identify and summarize youths' daily positive and negative identity‐related experiences; and (b) use insights from analyses to develop a conceptual model that integrates minority stress and resilience factors, socioecological systems, and within‐person temporal dynamics shaping the daily experiences of stress and resilience factors among LGBTQ+ youth.

#### Study context

Participants in the current study were situated within the developmental context of adolescence. Consistent with the developmental framework proposed by Sawyer et al. (2018), adolescence extends to 24 years of age and is characterized by distinct educational trajectories, role transitions, and ongoing neurobiological and psychosocial development. Notably, the onset of many mental disorders often occurs during adolescence or shortly thereafter, underscoring this stage as a critical period for mental health (Kessler et al., 2005, 2007). This phenomenon is particularly pronounced among LGBTQ+ youth, who experience disproportionately poorer mental health outcomes compared to their cisgender, heterosexual counterparts (Fish, 2020).

Daily diary data collected from LGBTQ+ youth must be interpreted within this developmental period, along with the broader socio‐political climate occurring during the study (March 2023–June 2024), as contemporaneous policies, public discourse, and sociocultural shifts are likely to influence youth's day‐to‐day experiences. The study's enrollment window was characterized by a sharp escalation in both the number and scope of legislative bans targeting the rights of LGBTQ+ populations, including access to gender‐affirming healthcare (Movement Advancement Project, 2023). Further, much of the legislation targeting the rights of LGBTQ+ youth has been concentrated in the U.S. Southeast, which includes this study's enrollment sites of Tennessee and Alabama (described below). Collectively, across both states, 76 anti‐LGBTQ+ bills were introduced during the enrollment period, and 23 were enacted (ACLU, 2023, 2024). Many of these laws directly limited the rights of LGBTQ+ youth, including bans on gender‐affirming care and requirements for schools to report students' gender identities, rendering the study's structural context highly stigmatizing.

## METHODS

### Participants and procedures

Community‐informed approaches were used in the study design phase, with the protocol informed by focus groups and interviews with LGBTQ+ youth and parents of LGBTQ+ youth (Clark, Phillips, et al., 2025). The study population included those ages 13–24 years old, who identified as LGBTQ+, reported at least mild depression and past‐year suicidal ideation during screening, and resided in Tennessee or Alabama (in‐depth details about the study protocol can be found in Clark, Phillips, et al., 2025). Although adolescence is a developmental period characterized by identity exploration, we recruited youth who self‐identified as LGBTQ+ to ensure participants could meaningfully interpret and respond to items assessing experiences of identity‐related minority stress and resilience factors. This was a prerequisite given the parent study's aim of examining associations between minority stress and suicidal ideation in daily life. Participants were recruited using convenience sampling through direct outreach to clinical and community settings, social media, and relevant email lists and listservs. A total of 99 individuals expressed interest in the study. Of those 99 individuals, 81 completed an eligibility screener. The final sample included 50 participants who consented to participate in the study. All study procedures were approved by the Vanderbilt University Medical Center Institutional Review Board. Informed consent was obtained from adult participants, and assent and parent consent were obtained from youth participants.

Consenting participants were enrolled in a smartphone‐based EMA protocol and completed three surveys per day (morning, afternoon, and evening) for 28 consecutive days via *MetricWire*. The present study focuses on data from the nightly diaries (administered between 8 and 10 pm in participants' local time zones), which included two open‐ended diary prompts in which participants qualitatively described the most positive and most negative events of their day (see Measures for additional details). Participants had two hours to complete the nightly survey, and they received up to 6 reminder notifications spaced approximately 15–30 min apart. Trained research assistants reviewed all negative diaries for indications of suicidal intent to assess whether a safety check‐in was needed, but none were reported across all nightly diaries (*N* = 1130).

The participants' mean age was 18.38 (SD = 3.02). The sample was predominantly White (*n* = 41, 82.0%), with smaller proportions identifying as Black/African American (*n* = 4, 8.0%), Asian (*n* = 2, 4.0%), multiracial (*n* = 2, 4.0%), or another race (*n* = 1, 2.0%). In terms of ethnicity, most participants identified as non‐Hispanic (*n* = 39, 92.0%), with a smaller subset identifying as Hispanic or Latinx (*n* = 4, 8.0%). Participants reported diverse gender identities. Approximately one‐third identified as girls or women (*n* = 17, 34.0%), while *n* = 6 (12.0%) identified as boys or men. Transgender identities were also represented, including trans boys or men (*n* = 11, 22.0%) and trans girls or women (*n* = 4, 8.0%). Additionally, a substantial portion of the sample identified with nonbinary and other gender‐diverse identities, including nonbinary (*n* = 13, 28.0%), genderfluid (*n* = 2, 4.0%), gender nonconforming (*n* = 2, 4.0%), genderqueer (*n* = 3, 6.0%), agender (*n* = 4, 8.0%), and other identities (*n* = 4, 8.0%). Regarding employment status, most participants were either full‐time students with part‐time work (*n* = 16, 32.0%) or unemployed students (*n* = 14; 28.0%). Most participants (*n* = 46, 92.0%) lived in Tennessee, with the remainder of the sample living in Alabama. Regarding participants' histories of suicidal thoughts and behaviors, 35 (70.0%) had seriously considered suicide, 35 (70.0%) had engaged in non‐suicidal self‐injury, and 17 (34.0%) had attempted suicide in their lifetimes.

### Measures

#### Daily diaries

In each nightly diary, participants responded either “Yes” or “No” to the following statement: “In the past 24 hours, I had a negative experience regarding my LGBTQ+ identity.” If they responded “Yes,” they were prompted to “Briefly describe one negative event or situation that you experienced in the last 24 hours that relates to your LGBTQ+ identity.” If they responded “No,” they were prompted to briefly describe a general negative event or situation. A parallel format was replicated in each nightly diary to assess positive daily events related to LGBTQ+ identity, or a general positive event or situation for participants who reported they did not have a positive LGBTQ+ experience that day. Only identity‐related nightly diaries were included in analyses.

### Analysis

In this manuscript, within‐person processes refer to processes occurring at the level of the individual (e.g., cognitive, emotional, or behavioral dynamics that unfold over time for a single participant), whereas between‐person processes refer to those that emerge across participants. While we adopted a qualitative rather than quantitative approach, we believe that these terms remain well‐suited to describe the varying levels of analysis we undertook.

#### Qualitative content analysis (QCA)

A qualitative content analysis (QCA) was conducted to systematically code diaries into predefined categories, enabling the identification, quantification, and interpretation of participants' positive and negative identity‐related experiences (Schreier, 2012; Hsieh & Shannon, 2005). QCA was considered the best fit for analysis, as data were often brief and frequently lacked detailed information, making traditional in‐depth qualitative methods less appropriate. This systematic approach to classifying and quantifying meaning within short, unstructured texts facilitated the identification and interpretation of themes and subthemes in the dataset. The QCA was conducted as a qualitative between‐subjects analysis, as it aimed to conceptualize factors that emerge across participants' identity‐related diaries. In other words, this analysis focused on identifying experiences of stress and strengths, with the emphasis being on identifying the presence/absence of themes across participants, rather than establishing temporal ordering across individual participants' responses.

We used an iterative, deductive approach to develop a coding structure. To classify identity‐related experiences within daily diaries, codes were drawn from the minority stress framework (Meyer, 2003) and its adolescent adaptation (Goldbach & Gibbs, 2017), as well as research focusing explicitly on resilience factors, such as the minority strengths framework (Perrin et al., 2020), Therefore, the coding structure incorporated frameworks that conceptualize resilience factors as a buffer against stress (Lee et al., 2024; Meyer, 2014), in addition to those that independently examine strengths within LGBTQ+ populations (Flynn et al., 2024; Perrin et al., 2020). To further contextualize the environments in which these experiences occur, the study also drew from prior research applying socioecological systems theory to LGBTQ+ populations (Bronfenbrenner, 1979; Clark et al., 2024; Hatzenbuehler & Pachankis, 2016; van der Star, 2024). Diaries were treated as individual units of analysis, and multiple codes could be applied to each diary.

To assess agreement across coders, we used Cohen's (1960) procedure for assessing interrater reliability (high interrater agreement: κ ≥ .60). During the initial phase of coding, half of the daily diaries were double‐coded by the first author and an undergraduate research assistant, who met regularly to discuss disagreement and adjust the codebook accordingly. The third and fourth authors joined these meetings to resolve any coding disagreements and refine the codebook where necessary. During the initial phase of coding, interrater reliability was high overall across most codes (κ = .79–1.00). After establishing high interrater reliability on all codes, the first author and the undergraduate research assistant split remaining diaries to code independently. After coding all diaries, code frequencies were quantified, and salient quotes were extracted.

#### Assessing sustained daily stress and strengths processes as they unfold within individuals

A post‐hoc qualitative analysis was conducted to further explore individual LGBTQ+ youth's identity‐related experiences within their day‐to‐day life. The post‐hoc analysis aimed to conceptualize processes occurring within an individual participant over time, focusing on the temporal ordering of stress and resilience factor experiences within daily diaries over the study period. Specifically, informed by previous longitudinal research with LGBTQ+ youth on stress temporality (i.e., the time course of stress exposure; Chang et al., 2024), the QCA coding frame was applied to within‐person data (i.e., diary entries from individual participants) to identify sustained experiences of minority stressors and resilience factors over the 28‐day study period. To be included in this analysis, participants had to have submitted at least two positive or two negative identity‐related diaries over the 28‐day study period to ensure that sustained minority stress or resilience factors across entries were possible. Participants' positive and negative diaries were assessed as separate units of analysis, given that they were assessed through different nightly questions.

A participant was considered to have experienced *sustained* minority stress if two or more nightly diaries shared a common “minority stress” QCA code and described similar contextual factors shaping the experience. For example, a participant's experience of being misgendered by a family member could be considered related to a subsequent argument with the same family member about wearing gender‐affirming garments, as both events are characterized by a lack of support for the youth's gender identity from the family member. In contrast, the experience of misgendering from a parent would not be considered related to an experience of misgendering from a teacher or coworker, as these events occur by different actors in different social contexts. Similarly, a participant was determined to have *sustained* experiences of resilience factors if two or more of their diaries shared a common “minority strengths” QCA code and similar context. Participants' diaries were double‐coded by the first and second author, who met throughout the coding process to discuss discrepancies. The senior author resolved any disagreements.

#### Using insights from analyses to develop the conceptual model

To develop the *Daily Identity‐Related Stress and Resilience Model*, we followed a systematic, data‐driven process that integrated findings from both the between‐subjects QCA and the within‐person analyses assessing sustained experiences of minority stress or strengths. The goal was to produce a conceptual visualization that reflects how minority stress and resilience factor processes co‐occur in LGBTQ+ youth's everyday lives, while remaining grounded in existing socioecological frameworks.

Model development proceeded in three stages. First, thematic patterns identified through the QCA were documented and reviewed by the research team. Second, findings from the within‐person analysis were examined for temporal patterns. Third, after identifying key constructs and relationships through our analyses, the integrated findings were mapped onto the three socioecological levels (intrapersonal, interpersonal, structural), consistent with van der Star (2024); time‐stable social, contextual, and identity factors were also considered to represent the broader chronosystem (Bronfenbrenner, 1979). The resulting visualization was iteratively refined by the research team to ensure conceptual clarity and alignment with both the empirical findings and the theoretical literature.

## RESULTS

### Qualitative content analysis (QCA)

Of the 1130 nightly diary entries, 349 (30.9%) endorsed an identity‐related experience and were included in analyses. Of these 349 identity‐related diaries, 108 (30.9%) were negative, and 241 (69.1%) were positive. Descriptions of all QCA codes can be found in Table 1. A summary of theme frequencies overall and across positive and negative diaries can be found in Table 2. Positive and negative identity‐related experiences for all themes are provided in Table S1. Salient codes and subthemes are described in detail below. To stay as true as possible to participants' own words and lived experiences, we have not edited spelling, capitalization, or grammar in any diary responses reported in the pages below.

**TABLE 1:** Qualitative content analysis code description.

Framework/code	Description
**Minority stress**	
Proximal minority stress	Internalized negative attitudes and values formed from exposure to external prejudice and discrimination targeting one's minority identity
Distal minority stress	External stressors experienced by LGBTQ+ individuals due to their minority status.
Structural stigma	Formalized systems, institutions, and organizations that constrain opportunities, resources, and wellbeing of LGBTQ+ populations.
**Minority strengths**	
Identity exploration	Individual actions or internal reflections related to identifying, pursuing, expressing, and/or affirming their LGBTQ+ identity taken to identity affirmation, outness, expression
Community connectedness and engagement	Expressing or experiencing identification, affiliation, and connection with the LGBTQ+ community
External validation	External validation or affirmation of participants' LGBTQ+ identities
Structural supports	Formalized systems, institutions, and organizations that bolster opportunities, resources, and well‐being of LGBTQ+ populations.
**Socioecological context**	
Individual level	Individual experiences that directly affect thoughts, emotions, behaviors, or well‐being, especially internal processes and subjective responses
Interpersonal level	Interactions and relationships between individuals within their social environment
Vicarious experiences	Experiences observed in others that may influence individual attitudes, behaviors, and psychological states.
Friends/Romantic partners	Experiences with close peer relationships, including friendships, crushes, and romantic partners
Family	Experiences involving family, including romantic partners
Professional/Public interactions	Professional and public encounters in both structured environments and casual public interactions
Online interactions	Exchanges and relational experiences that individuals have through digital platforms
Structural level	How society, organizations, and institutions influence opportunities, behaviors, and access to resources and roles
Institutional influences	Societal institutions such as education, religious organizations, law/policy, and healthcare on individual
Media	Media exposure, engagement, and representation

**TABLE 2:** QCA frequency table.

Theme/Subtheme	Total IR diaries (*N* = 349)	Positive IR diaries (*n* = 241)	Negative IR diaries (*n* = 108)
	*n* (%)	*n* (%)	*n* (%)
Proximal minority stress	38 (10.9)	7 (2.9)	31 (28.7)
Distal minority stress	109 (31.2)	16 (6.6)	93 (86.1)
Structural stigma	30 (8.6)	3 (1.2)	27 (25.0)
Identity exploration	47 (13.5)	30 (12.4)	17 (15.7)
Community connectedness and engagement	86 (24.6)	78 (32.4)	8 (7.4)
Structural supports	45 (12.9)	44 (18.3)	1 (0.9)
Individual level		19 (7.9)	19 (17.6)
Interpersonal level			
Vicarious experiences	34 (9.7)	11 (4.6)	23 (21.3)
Friends/Romantic partners	142 (40.7)	127 (52.7)	15 (13.9)
Family	41 (11.7)	27 (11.2)	14 (13)
Professional/Public interactions	119 (34.1)	73 (30.3)	46 (42.6)
Online Interactions	35 (10)	11 (4.6)	24 (22.2)
Structural level			
Institutional influences	42 (12)	19 (7.9)	23 (21.3)
Media	42 (12)	25 (10.4)	17 (15.7)
External validation	81 (23.2)	79 (32.8)	2 (1.9)

#### Minority stress

In negative diaries, proximal minority stress (*n* = 31, 28.7%) regularly described experiences of identity concealment, gender dysphoria, and anticipated stigma. Although some diaries described experiences only characterized by proximal minority stress (i.e., *“felt disgusted with myself for being trans”*), proximal minority stress was often described in context with distal and structural stressors. Distal minority stress was described in the majority (*n* = 93, 86.1%) of negative identity‐related diaries and captured a variety of discriminatory experiences, including homophobic language, misgendering, and social rejection. One participant shared, “*A friend that I met at a previous job told me about times when our coworkers made fun of me for how I present and identify. It hurts to hear about other adults talking about you this way.”* Many negative diary descriptions of structural stigma (*n* = 27, 25%) referred to formalized restrictions limiting access to healthcare (e.g., gender‐affirming care bans), public accommodations (e.g., bathroom bans), freedom of speech and expression (e.g., drag bans), and other civil rights (e.g., religious exemptions that allow a person to deny the solemnization of a same‐sex marriage). Structural stigma in participants' nightly diaries was discussed within local, national, and even global context. In addition to legislation, diaries that discussed structural stigma also captured anti‐LGBTQ+ norms that participants were exposed to during daily interactions within their faith communities (e.g., *“I was at church and the priest had a homily about how men and women are meant for each other”*). Some diaries discussed structural stressors within the larger context of other forms of oppression, including racial discrimination (e.g., *“kept seeing news about tennessee trying to pass a law where whoever is marrying you can deny you if they want to* (*ex. lgbtq, race, etc)”*).

While infrequent, some positive identity‐related diaries also captured experiences of proximal (*n* = 7, 2.9%) and distal (*n* = 16, 6.6%) minority stress and structural stigma (*n* = 3, 1.2%). These diaries include positive experiences contextualized by gender dysphoria (e.g., “*I usually hate pictures of myself because I always look too masculine…but I kinda liked the random pictures [my friends] took. It felt like I looked a little more like myself”*), family nonacceptance (e.g., *“my great grandma took me to cracker barrel and the waiter had a pride safe space pin. it made me feel a little better because my extended family isn't very supportive”*), and anti‐LGBTQ+ policies (e.g., *“Talked to someone about all the bills being passed and they reassured me people support me”*). Taken together, these examples demonstrate how some positive moments of resilience and hope can occur even within the broader context of minority stress experiences that frequently permeate the daily lives of LGBTQ+ youth.

#### Minority strengths

In positive diaries, community connectedness and engagement characterized participants' sense of connection to the LGBTQ+ community through participation in formal (e.g., attending Pride celebrations, student organizations, support/social groups, etc.) and informal (e.g., discussions about identity among LGBTQ+ peers, advice giving, etc.) LGBTQ+ spaces. While some experiences of community connectedness and engagement described connection to the LGBTQ+ community through interactions with individual members of the community, such as friends/peers, romantic partners, and neighbors (e.g., “*[I] had dinner with two queer friends and we talked about our queer experiences”*), others highlighted the connection with the broader community (e.g., “*It was so nice to see so many allies and members of the LGBTQIA community come together”*). One positive diary entry also described community connectedness interacting with other axes of identity‐affirmation (e.g., *I am facilitating my school's queer & Asian group with my friend and had a nice email exchange with the director of LGBTQI life*).

External validation captured a variety of experiences, ranging from minor interpersonal acknowledgements (e.g., “*someone complimented my lgbt shirt I wore today”*) to overt gestures of institutional support (e.g., “*My [boss] wants to have a pride event… [I]t's nice to know that I have a LGBTQ safe work environment and can help keep it that way for others”*). Participants also named a variety of structural supports (*n* = 44, 18.3%) that provided formalized access to resources, programming, or advocacy for LGBTQ+ populations. Structural supports fostered opportunities for connection and engagement with the LGBTQ+ community through organized events and institutional resources. Examples included university‐hosted events, such as lavender graduations and pride celebrations, as well as LGBTQ+ proms organized by community groups. Named structural supports also encompassed university identity centers and organizations (e.g., Office of LGBTQI+ Life), gender‐affirming healthcare (e.g., insurance coverage), progressive faith communities (e.g., affirming pastors), and community organizations (e.g., LGBTQ+ nonprofits).

While community connectedness and engagement and external affirmation were most common in positive identity‐related diaries, participants also occasionally evoked these themes in negative entries. For example, participants mentioned engaging with LGBTQ+ people, while also encountering experienced and/or anticipated rejection from people outside the group. One participant's negative identity‐related diary read, “*Some girls were making fun of a group I was in…[that] consisted of a lot of openly out LGBTQ+ people so it made me self‐conscious that that was what they were talking and laughing about.”* Similarly, examples of external validation from negative diaries illustrated its overlapping complexity with distal minority stressors—specifically, experiences that simultaneously involved validation and invalidation. One participant shared, *“people assumed that I don't know what it is like to be pregnant because I am male passing. not necessarily negative because of my [LGBTQ] identity, but because people assume that I am not [LGBTQ].”* This entry captures a nuanced experience in which being perceived as male validated the participant's gender presentation while simultaneously overlooking their reproductive capacity as a transgender individual. Together, these negative diaries highlight the complexities embedded in day‐to‐day experiences of community connectedness and external validation.

#### Socioecological context

Across diaries, participants referred to varied, interconnected socioecological levels characterizing experiences of stress and strengths in everyday life. Intrapersonal identity‐related experiences were more prevalent among negative diaries (*n* = 19, 17.6%) than in positive diaries (*n* = 19, 7.9%). Regarding negative intrapersonal presentations of proximal minority stress, internalized stigma and gender dysphoria were commonly described. Among positive diaries, intrapersonal experiences often described internal reflections related to identity affirmation (e.g., *“I thought about my collection of trans artwork, which makes me happy and feel seen”*) and exploration (e.g., *“realized [I'm] probably demiromantic”*).

Among diaries coded at the interpersonal level, most positive diaries described events that occurred between friends or romantic partners (*n* = 127, 52.7%). Many positive diaries described experiences of validation and community connectedness alongside friends and romantic partners (e.g., *“My friends and I went to Target and we saw the pride merch. It got us all really excited for pride in a month”*). Others referenced distal minority stressors unfolding alongside positive interpersonal experiences (e.g., *“Talked to my girlfriend and made plans to go out again soon! Knowing that this is a stressful environment for queer folks, it's always nice to just make plans with her and pretend there's no concerns”*).

Negative diaries more commonly described public or professional interactions (e.g., with teachers, bosses, strangers; *n* = 46, 42.6%). For instance, one participant shared, “*My girlfriend and I were holding each other… in the back of her car parked at a public park and a guy on a motorcycle started driving around us, [revving] his engine and yelling at us until we felt unsafe and left.”* This example illustrates how an interpersonal interaction with a romantic partner—a context commonly associated with positive diaries—can simultaneously serve as a backdrop for a negative encounter with a stranger in public.

At the structural level, institutional contexts, including K‐12 and higher education, religion, and local and national politics, were frequently described in negative diaries. For example, one participant shared that they, “*argued with someone on Instagram regarding the 2 anti‐trans laws that passed today in [their state].”* While LGBTQ+ youth may have infrequent or isolated direct interactions with structural contexts, such as healthcare settings, in daily life, they described regular structural exposures through media, including television, social media, and video games. Media exposure was more frequently described in negative diaries (*n* = 17, 15.7%) than positive diaries (*n* = 25, 10.4%). Among negative diaries, media exposure often involved daily exposure to anti‐LGBTQ+ legislation and violence against LGBTQ+ community members (e.g., “*I saw some upsetting news online about people in my community being harassed. It made me upset and made me worried for my own safety”*). Exposure to structural stigma (e.g., news of LGBTQ+ discriminatory policies) also emerged in positive diaries, often through discussion of associated artistic events or protests. One salient example described attending a concert where the artist condemned state‐level anti‐LGBTQ+ legislation and featured drag performances, visibly rallying community support, with the participant noting, “*The best part was the unanimously supportive reaction of the crowd”*.

Taken together, positive and negative identity‐related diaries illustrated interactions across socioecological levels, offering insight into how structural‐level factors shape day‐to‐day intrapersonal and interpersonal experiences. At the intrapersonal level, participants shared internal reflections influenced by structural‐level stressors (e.g., “*Realizing I only have a couple weeks until my identity is illegal*”) and supports (e.g., “*realized my two year anniversary of testosterone is coming up soon!*”). Structural‐level stressors also shaped participants' positive and negative interpersonal experiences, particularly in online contexts, including those that were both positive (e.g., "*talked* *to this lovely lady online, gave support to me for everything happening in the state"*) and negative (e.g., "*my* *uncle posted on Facebook that he wants to know if the devil has horns [because] I will be seeing them"*). This pattern underscores the interconnected relationship between LGBTQ+ youth and the broader social systems in their everyday life.

### Sustained daily stress and strengths processes: Qualitative within‐person analysis

On average, participants submitted more positive identity‐related diaries (*M* = 5.11, SD = 4.92) than negative identity‐related diaries (*M* = 2.4, SD = 2.38) over the 28‐day study period. Among the 32 participants who submitted at least two negative identity‐related diaries, 17 (53.1%) participants described sustained experiences of minority stress across the study period. Sustained distal stressors, including structural stigma, were more common than sustained proximal stressors, and sustained references to distal minority stress frequently occurred in interpersonal contexts, including workplace and familial interactions.

For example, one participant submitted three negative diaries describing ongoing conflict with their parents regarding non‐acceptance of their transgender identity, illustrating sustained distal minority stress across the study period. The first diary addressed conflict broadly around, *“whether or not trans healthcare is ‘experimental’”*; however, subsequent entries more directly engaged the participant's identity development and social transition. A later diary shifted from recounting a specific conflict to expressing accumulated frustration over their parents' prolonged nonacceptance: *“I'm still at odds with my parents over them calling themselves supportive just because they haven't thrown me out of the house. That's not even acceptance – that's tolerance.”* This participant's sustained experience of familial non‐affirmation illustrates how minority stress moves fluidly across socioecological levels. Indeed, these familial interactions were not isolated but rather unfolded within the participant's individual identity development and the broader structural stigma present within their regional context.

Among the 42 participants who submitted two or more positive identity‐related diaries, 30 (71.4%) reported sustained experiences of resilience factors, meaning that at least one experience of minority strength described in a positive identity‐related diary was reflected in a subsequent entry. Among these sustained positive identity‐related experiences, the most common resilience factor was community connectedness and engagement, which was most frequently experienced alongside friends and romantic partners.

For instance, one participant submitted several positive diaries documenting a gradual shift in their mother's acceptance of their romantic relationship, illustrating an evolving trajectory of parental affirmation. The participant first identified a shift in their mother's viewpoint, “*[…]My mom is starting to become more accepting of my girlfriend and my sexuality”*, followed by a milestone of bringing their partner home, “*My girlfriend was allowed to come over, and then my mom asked her to stay for dinner*,” and, later, a fuller expression of acceptance, “*My girlfriend came over to my house… and my mom was really accepting of her*.” Submitted across the 28‐day period, these entries depict not only a sustained sense of maternal validation, but a positive trajectory of parental acceptance shaped by the continued efforts of both the participant and their partner over time. At the within‐person level, sustained experiences of resilience factors across positive diaries offer nuanced insight into the short‐term trajectories of minority strengths in everyday life, revealing how past experiences cumulatively shape a sense of support, with each entry building on and informing the next. Overall, these qualitative within‐person analyses of identity‐related diaries illuminate the ongoing, day‐to‐day processes of minority stress and resilience factors over a month‐long period in the life of LGBTQ+ youth in the southeastern United States.

### Daily identity‐related stress and resilience model

Based on insights from qualitative analyses of daily identity‐related experiences, we developed the *Daily Identity‐Related Stress and Resilience Model* (Figure 1), extending existing socioecological frameworks of minority stress and resilience factors to capture within‐person dynamics relevant to daily life for LGBTQ+ youth. Using a three‐day snapshot of within‐person experiences (Day *t*−1, Day *t*, Day *t* + 1), identity‐related stress (IRS) and identity‐related resilience (IRR) are depicted as simultaneously unfolding across three socioecological levels (intrapersonal, interpersonal, structural) within each day, consistent with van der Star (2024). The central day (Day *t*) illustrates the full range of exemplar experiences at each level: at the structural level, IRS may include seeing news reports about anti‐LGBTQ+ laws or learning about a new school policy banning same‐gender partners at school dances, while IRR may include receiving insurance approval for gender‐affirming medical care or attending an affirming faith ceremony; at the interpersonal level, IRS may include being misgendered by a coworker or a family member sending a transphobic online message, while IRR may include a parent gifting a gender‐affirming clothing item (e.g., a dress) or sharing advice with an LGBTQ+ peer; and at the intrapersonal level, IRS encompasses experiences such as gender dysphoria and internalized stigma, while IRR centers on gender euphoria, identity pride, and identity expression. The sequential arrangement of days (Day *t*−1 ➔ Day *t* ➔ Day *t* + 1) conveys that these multilevel processes unfold over time, with experiences on any given day embedded within, and potentially shaped by, those of preceding days. An arrow from Day *t*−1 ➔ Day *t* + 1 further highlights that experiences on prior days may influence future days beyond the immediate next day.

**FIGURE 1:**
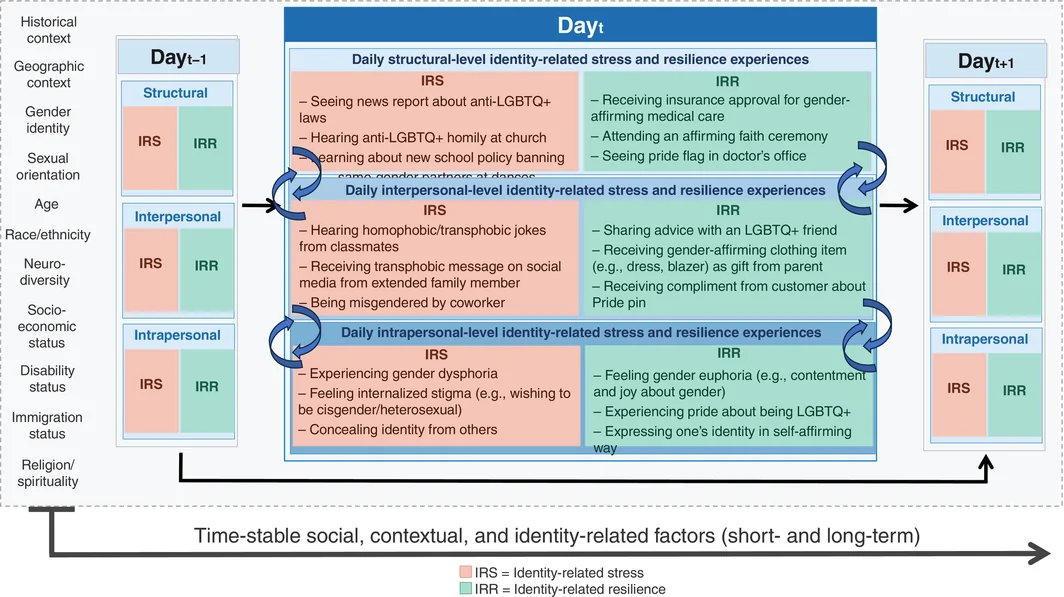
Daily identity‐related stress and resilience model. Developed from daily diary data from LGBTQ+ youth in the Southeastern US.

We further situate daily within‐person experiences within a broader chronosystem (Bronfenbrenner, 1979; van der Star, 2024) to illustrate how larger temporal scales, including historical context and the developmental life course, shape daily stress and resilience processes. The time‐stable social, contextual, and identity‐related factors represented in the left panel—including historical context, geographic environment, gender identity, sexual orientation, race/ethnicity, age, neurodiversity, socioeconomic status, disability status, immigration status, and religion/spirituality—do not typically fluctuate meaningfully over very short time intervals (i.e., days), yet continuously shape how individuals encounter and navigate identity‐related experiences at each socioecological level in daily life. The bracket connecting this panel to the horizontal arrow, labeled *Time‐stable social, contextual, and identity factors (short and long term)*, visually communicates that these factors operate as a persistent contextual backdrop across both the brief day‐to‐day window depicted in the model and longer developmental and historical timescales.

## DISCUSSION

This study explored processes of minority stress and resilience factors as they unfolded in the daily lives of LGBTQ+ youth living in the U.S. Southeast during a period of heightened structural stigma. Participants submitted nearly twice as many positive identity‐related diaries as negative ones, a pattern that is notable given the regional and legislative context in which the data were collected. Descriptions of structural stigma were common and frequently referenced specific legislation, particularly anticipated and enacted restrictions on gender‐affirming care access. This pattern is consistent with the demographic composition of our sample, which included a substantial proportion of transgender and gender‐diverse participants, many of whom were under 18 and, therefore, directly affected by the legislative restrictions. Below, we discuss findings from the research in conjunction with the extant literature and offer opportunities for future research and intervention.

### Co‐occurrence of stress and resilience in daily life

The daily diary data revealed that minority stress and resilience factors did not operate as mutually exclusive or sequentially ordered processes, but rather as contemporaneous experiences that frequently intersected within a single day and across socioecological levels. This finding challenges prior theoretical work that has leveraged the minority stress framework (Meyer, 2015), primarily through a deficit‐oriented lens. Instead, our data suggest that stress and resilience factors co‐occur dynamically—participants described moments of affirmation and community connectedness that unfolded alongside, or even in direct response to, experiences of stigma. For example, participants described interpersonal validation from peers or partners that occurred within the broader context of structural‐level stressors, and positive intrapersonal experiences, such as identity pride, that emerged alongside ongoing experiences of gender dysphoria or family non‐acceptance. These findings extend upon the minority strengths model (Perrin et al., 2020) and qualitative research documenting sources of queer and trans joy in daily life (Flynn et al., 2024), by demonstrating that the co‐occurrence of stress and resilience factors is visible at the within‐person, day‐level.

This co‐occurrence has implications for how researchers conceptualize and measure minority stress and resilience factors in intensive longitudinal designs. Studies that assess stress and resilience factors separately, or that treat identity strengths as simply the absence of distress, may obscure the simultaneous presence of both processes within a given day. Our findings suggest that future daily diary studies and intensive longitudinal research (e.g., EMA) would benefit from explicitly assessing both stress and resilience processes within the same reporting window, in addition to using analytic approaches able to capture their interaction over time and its effects on mental health outcomes.

### Social context in the daily lives of LGBTQ+ youth

The salience of social context in shaping both positive and negative identity‐related experiences was a prominent feature of participants' nightly diaries. This emphasis aligns with prior minority stress research highlighting the importance of social contexts—including family, peers, school, and community—among LGBTQ+ adolescents (Goldbach & Gibbs, 2017), as well as resilience research underscoring the protective role of social connection and community engagement (Flynn et al., 2024). Extending this work, our findings illustrate how broader socioecological dynamics can simultaneously generate both stress and resilience factors within the same interpersonal context. For instance, participants described how engagement with LGBTQ+ community spaces and events—including Pride celebrations, student organizations, and affirming faith communities—functioned as sources of connection and validation, even though these spaces sometimes exposed participants to stigma or anticipated rejection from non‐LGBTQ+ people.

The interpersonal level emerged as particularly central in both positive and negative diaries, with friends, romantic partners, and family members featuring prominently across entries. Positive interpersonal experiences, particularly with peers and partners, were the most frequently described form of resilience factors, while negative interpersonal experiences in public and professional settings were among the most common stressors. This pattern underscores the importance of close relationships as a primary site of both vulnerability and protection for LGBTQ+ youth. It also points to the potential value of interventions, such as gender‐sexuality alliances (GSA), that strengthen interpersonal support networks alongside structural advocacy efforts (Pearson et al., 2026; Toomey & Russell, 2013).

### Structural stigma in everyday life

A distinctive feature of participants' narratives was the frequency and specificity with which structural‐level stigma appeared in their daily experiences. Even in short diary entries, participants commonly described specific legislative developments, anti‐LGBTQ+ policies, and institutional barriers to affirmation. Media exposure, including via social media, news, and online interactions, served as a primary conduit through which structural stigma entered participants' daily lives. The finding is consistent with prior EMA research linking exposure to structural stigma, such as anti‐LGBTQ+ news, with within‐person increases in suicidal ideation among LGBTQ+ youth (Clark et al., 2024). Importantly, structural stressors did not only remain at the structural level; indeed, participants' diaries frequently illustrated how policy‐level experiences reverberated through interpersonal interactions and intrapersonal processes, producing downstream effects on participants' sense of safety, identity, and well‐being on a given day. This cross‐level transmission of structural stigma reinforces the importance of multilevel conceptualizations of minority stress (Hatzenbuehler & Pachankis, 2016; van der Star, 2024) and highlights the need for structural interventions, including policy change and institutional advocacy, as essential complements to individually focused mental health supports (Clark, Phillips, et al., 2025; Clark, Rajah, et al., 2025).

At the same time, structural supports, including university LGBTQ+ centers, gender‐affirming healthcare access, and affirming faith communities, played a meaningful role in participants' positive diary entries, often serving as scaffolding for community connection and interpersonal validation.

### Temporality and within‐person continuity in positive and negative identity‐related experiences

Qualitative within‐person analyses revealed that both minority stress and resilience factors showed meaningful continuity across days, with the majority of participants who submitted multiple identity‐related diaries describing sustained experiences of either stress or resilience factors over the 28‐day study period. Sustained stress was more commonly rooted in distal stressors—particularly interpersonal experiences within family and workplace contexts—than in proximal stressors, suggesting that the persistence of minority stress over time may be more closely tied to ongoing relational dynamics than to momentary intrapersonal states. However, this finding may be partially attributable to the wording the research team utilized for daily diaries, as participants were prompted to describe an event rather than an internal reflection. Therefore, participants may have been more likely to report interpersonal—rather than intrapersonal—experiences. Sustained resilience factors most commonly involved community connectedness and engagement, with participants describing ongoing or deepening connections with LGBTQ+ peers, partners, and community spaces over the course of the study period.

These within‐person patterns speak to the importance of considering temporal dynamics in minority stress and resilience research. As argued by Earnshaw et al. (2022) and Rivas‐Koehl et al. (2023), time is not a monolithic backdrop to LGBTQ+ health but rather a dimension that operates across multiple scales—from historical and generational time to the granular, day‐to‐day unfolding of stress and resilience factors within an individual's life. Our analysis provides preliminary evidence that experiences of stress and resilience accumulate and evolve over short time periods, with each day's experiences potentially shaping the context in which subsequent days are encountered. This temporal continuity is not yet well captured by existing minority stress or strengths frameworks, most of which have been developed with cross‐sectional or between‐person data in mind. Adopting chronicity‐informed perspectives, this finding represents an important direction for future theoretical and empirical work.

Future such research may be informed by the *Daily Identity‐Related Stress and Resilience Model* (Figure 1) which integrates multilevel socioecological theory along with an emphasis on within‐person temporal dynamics and co‐occurrences of stress and resilience factors. In situating daily experiences within a broader chronosystem (Bronfenbrenner, 1979; van der Star, 2024), our proposed model acknowledges that the day‐level processes it depicts are themselves embedded within longer developmental and historical timelines. The enrollment period for this study, characterized by unprecedented legislative attacks on LGBTQ+ rights in the U.S. Southeast, provides a vivid illustration of how historical time shapes the structural context within which daily experiences unfold. Future applications of the model should attend to the specific historical and regional contexts in which LGBTQ+ youth are situated, as these broader forces are not incidental to daily experience but constitutive of it. This is consistent with seminal developmental science conducted outside of the scope of LGBTQ+ youth, including García Coll et al.'s (1996) integrative model of developmental competencies in minority children, which established that the well‐being of minoritized youth cannot be understood apart from the social structures and historical contexts in which their development is embedded.

### Limitations

Several limitations warrant consideration. First, our analyses do not account for developmental differences across the wide age range of the sample (13–24 years), as experiences of stress, coping, and resilience factors may vary considerably across early adolescence, late adolescence, and emerging adulthood. Future research should examine how developmental stage interacts with minority stress processes to identify age‐specific risk and protective factors.

Second, although a waiver of parental consent was sought, institutional review board approval was not granted, requiring parental consent for participants under the age of 18. Consequently, participation was restricted to minors who had disclosed their identities to their parents. The methodological implications of these recruitment constraints are discussed in greater detail elsewhere ([blinded for review]). Future research should continue to pursue waivers of parental consent to facilitate the inclusion of LGBTQ+ adolescents while maintaining ethical protections (Mustanski, 2011).

Third, although the sample was diverse in sexual orientation and gender identity, it was predominantly White, limiting our ability to capture intersectional minority stress and resilience processes. The diary prompt also focused on LGBTQ+ identity, without explicitly eliciting intersectional experiences—a notable gap given that nearly a third of Black transgender youth live in states with gender‐affirming care bans (Movement Advancement Project, 2023) and that minority stressors are frequently attributed to multiple minoritized identities simultaneously (Mereish et al., 2021). Future research should integrate intersectional frameworks into EMA and daily diary methodologies, including mixed methods approaches, such as those leveraged by Jackson et al. (2021).

Finally, while qualitative daily diaries offered rich contextual insight, they are limited in their ability to model temporal dynamics and within‐person effects with the precision of quantitative approaches (Spencer et al., 2021). Within‐person analyses provided preliminary evidence of cross‐day continuity; importantly, however, the daily diary prompt was not explicitly designed to test carryover effects or lagged associations. Future research should consider leveraging prompts that more directly capture chronicity (e.g., “Were you impacted by this event yesterday?”), in addition to integrating novel approaches that could better capture the temporal dynamics described in the *Daily Identity‐Related Stress and Resilience Model*, such as Thematic Trajectory Analysis (Spencer et al., 2021).

## CONCLUSION

Using qualitative daily diary data from an intensive longitudinal study, we examined how minority stress and resilience factors unfolded in the everyday identity‐related experiences of LGBTQ+ youth at elevated risk for suicide in the U.S. Southeast. Between‐person analyses revealed that minority stress and resilience factors mutually shaped daily identity‐related experiences across structural, interpersonal, and intrapersonal levels, co‐occurring within single days rather than operating as separate or opposing processes. Qualitative within‐person analyses further demonstrated that both stress and strengths showed meaningful continuity across days, with repeated exposures shaping the trajectory of subsequent experiences. Together, these findings informed the development of the *Daily Identity‐Related Stress and Resilience Model*—a conceptual framework that integrates minority stress and strengths theory, socioecological systems, and within‐person temporal dynamics to more fully capture how LGBTQ+ youth navigate identity‐related experiences in daily life. We hope this model serves as a foundation for future research that attends to the temporal and multilevel complexity of the LGBTQ+ adolescent experience, along with inspiring intervention developments targeting both stressors and strengths in LGBTQ+ youth's everyday lives.

## AUTHOR CONTRIBUTIONS

**Kaitlyn Phillips:** Conceptualization; writing – original draft; methodology; visualization; writing – review and editing; formal analysis; data curation; project administration. **Alexandros Nikolaidis‐Konstas:** Formal analysis; visualization; writing – review and editing; writing – original draft. **Eric Layland:** Supervision; conceptualization; methodology; visualization; writing – review and editing. **Kirsty Clark:** Conceptualization; visualization; funding acquisition; supervision; writing – review and editing; data curation; methodology; project administration; investigation.

## FUNDING INFORMATION

This work was funded by the National Institute of Mental Health of the National Institutes of Health under award number K01MH125073 (PI: Clark). The content is solely the responsibility of the authors and does not necessarily represent the official views of the National Institutes of Health.

## CONFLICT OF INTEREST STATEMENT

The authors declare no conflicts of interest.

## ETHICAL APPROVAL

Ethical approval for this study was obtained from the Vanderbilt University Medical Center institutional review board on Jan 30, 2023 (IRB#222289).

## PATIENT CONSENT

Informed consent was obtained from adult participants, and assent and parent consent were obtained from youth participants.

## Supporting information


**Table S1.** Example quotes.

## Data Availability

De‐identified data supporting the findings of this study are available from the corresponding author upon reasonable request and completion of a data use agreement. The data are not publicly available due to privacy and ethical restrictions.
