# Supplementary material for: Qualitative development of the daily identity‐related stress and resilience model using daily diaries from LGBTQ+ youth in the southeastern United States
Source: J Res Adolesc. 2026 Oct 20;36(4):e70289. doi: 10.1111/jora.70289 (PMC13681246; doi:10.1111/jora.70289)
Supplement: Supplementary file 1 [file JORA-36-0-s001.docx]

Table S1. Example Quotes

| **Theme/**  **Subtheme** | ***Positive IR Examples*** | ***Negative IR Examples*** |
| --- | --- | --- |
| Proximal Minority Stress | "I got to spend time with my girlfriend and we went on a hike. We were allowed to hold hands because no one else was around, which was nice." | "Some girls were making fun of a group I was in today and it made me anxious because I was imagining all the things they were saying. The group consisted of a lot of openly out LGBTQ+ people so it made me self conscious..." |
| Distal Minority Stress | "...listening to a close friend's parents - who I'm closeted to - discuss the current politicized media fracas about Dylan Mulvaney partnering with BudLight and Nike..., they were surprisingly supportive and well educated about transness for people of their age and demographic." | “I had negative reactions from people I thought would be accepting when sharing how I felt about an LGBTQ+ issue.” |
| Structural Stigma | "Talked to someone about all the bills being passed and they reassured me people support me" | "Watching the news, and continuing to hear about the numerous anti-trans laws and how some people feel they need to be pushed harder to “get the gays off the streets” |
| Identity Exploration | "Punk rock flea market! Being loudly and openly queer in public and supported vocally by everyone there." | “I witnessed some people close to me misgendering someone. It made me feel like I could never identify as nonbinary and have it be respected.” |
| Community Connectedness and Engagement | "I went out to get food with a local group that I was in last year and it was great getting to be gay with all the other people my age" | "I had to deal with a gay person on twitter claim they’re not gay, and that queer is a slur, and that I should feel bad for using a slur to describe myself" |
| Structural Supports | "i got my hysterectomy approved by insurance!! im really excited, this is a huge part of my gender affirming care plan" | "I posted pictures from the lgbtqia graduate ceremony and some people unfollowed me [because] of it. " |
| Individual Level | "I wore a skirt in public for the first time in a while and bought some gender affirming clothes." | "felt disgusted with myself for being trans" |
| Interpersonal level |  |  |
| Vicarious experiences | "I went to a drag show and saw other queer people, including an extremely open nonbinary person. it was refreshing to see everyone being queer and having such a good time together" | "I witnessed some people close to me misgendering someone. It made me feel like I could never identify as nonbinary and have it be respected." |
| Friends/Romantic Partners | "I participated in the lavender graduation ceremony for lgbtqia graduates. I had friends I knew there and felt very celebrated and loved" | "I was talking to my ex (cis male) about why we broke up and he said one of the reasons was that he didn’t know if one day I would want to date a girl instead of him. or that I would no longer want to date a guy one date. this made me really sad and misunderstood" |
| Family | "My mom casually referred to me as "Mr"" | "my uncle posted on Facebook that he wants to know if the devil has horns [because] I will be seeing them " |
| Professional/Public Interactions | "i got gendered properly in the drive thru today!! so exciting every time" | "Got misgendered several times at the store today " |
| Online Interactions | "I shared a selfie on discord and received compliments" | "People making weird and transphobic comments on one of my pride post on instagram so I turned off comments on the post." |
| Structural Level |  |  |
| Institutional Influences | "Lativa's head came out as openly gay. Recently I was also reading about Ireland's LGBTQ culture and how accepting they are." | "I was at church and the priest had a homily about how men and women are meant for each other. That made me feel unaccepted by the church." |
| Media | "I discovered a new singer I really enjoy... she shares a lot of backgrounds and identity with me. I'm never one for celebrity worship, but it's nice to resonate with her music." | "i wanted to have fun and mess around with like an ai chatbot, but it started being super homophobic and wouldn’t stop so it kinda just ruined the experience:(" |
| External Validation | "My cousin is marrying a woman and my nana said she didn't care as long as she takes care of her...her reaction settled my nerves for when I do decide to tell her. " | " people assumed that I don’t know what it is like to be pregnant because I am male passing. not necessarily negative because of my lgbtq+ identity, but because people assume that I am not lgbtq+" |
